# Dichloroacetate and Salinomycin Exert a Synergistic Cytotoxic Effect in Colorectal Cancer Cell Lines

**DOI:** 10.1038/s41598-018-35815-4

**Published:** 2018-12-10

**Authors:** Aistė Skeberdytė, Ieva Sarapinienė, Jan Aleksander-Krasko, Vaidotas Stankevičius, Kęstutis Sužiedėlis, Sonata Jarmalaitė

**Affiliations:** 1 Institute of Biosciences, Life Sciences Center, Vilnius University, Saulėtekio al. 7, LT-10257 Vilnius, Lithuania; 2 Institute of Cardiology, Lithuanian University of Health Sciences, Sukilėlių pr. 15, LT-50162 Kaunas, Lithuania; 3 National Cancer Institute, Santariškių g. 1, LT-08660 Vilnius, Lithuania; 4 Institute of Biotechnology, Life Sciences Center, Vilnius University, Saulėtekio al. 7, LT-10257 Vilnius, Lithuania

## Abstract

In the present study, we examined a hypothesis that dichloroacetate, a metabolic inhibitor, might efficiently potentiate the cytotoxic effect of salinomycin, an antibiotic ionophore, on two human colorectal cancer derived cell lines DLD-1 and HCT116. First, we performed a series of dose response experiments in the 2D cell culture by applying mono- and combination therapy and by using the Chou-Talalay method found that salinomycin in combination with dichloroacetate acted synergistically in both cell lines. Secondly, in order to recapitulate the *in vivo* tumor architecture, we tested various doses of these compounds, alone and in combination, in the 3D multicellular spheroid culture. The effect of combination of dichloracetate and salinomycin on multicellular spheroid size was stronger than the sum of both monotherapies, particularly in HCT116 cells. Further, we demonstrate that the synergistic effect of compounds may be related to the inhibitory effect of dichloroacetate on multidrug resistance proteins, and in contrast, it is not related to dichloroacetate-induced reduction of intracellular pH. Our findings indicate that the combination therapy of salinomycin and dichloroacetate could be an effective option for colorectal cancer treatment and provide the first mechanistic explanation of the synergistic action of these compounds.

## Introduction

Colorectal cancer (CRC) is the third most commonly diagnosed cancer in both men and women^1^. Despite significant reductions in overall colorectal cancer incidence and mortality, a dramatic number of nearly 1.4 million new cases are diagnosed every year. CRC is usually treated surgically in combination with radiation and/or chemotherapy, depending on tumor location and disease progression^2,3^. Standard approved chemotherapy regimens for CRC patients are FOLFOX, which includes folinic acid, 5-fluorouracil (5-FU), and oxaliplatin, and FOLFIRI where oxaliplatin is replaced by irinotecan^4^. Considering the fact that majority of the treated tumors eventually develop resistance to 5-FU, a novel therapeutic approach or new combination treatments are of crucial importance^5,6^. Combination therapy is the cornerstone of cancer treatment. The simultaneous application of cytotoxic drugs potentiates their efficacy compared with monotherapy because it targets the key pathways in a synergistic or an additive manner. Such therapy is likely to diminish drug resistance, while simultaneously providing cytotoxic benefits, such as inhibition of tumor growth, decrease of cancer stem cell population, reduction of metastatic potential, and induction of apoptosis^7^.

Salinomycin is a monocarboxylic polyether ionophore that has been discovered in high throughput screening as a potential anti-cancer drug selectively targeting breast cancer stem cells^8^. This finding resulted in numerous experiments performed on other types of cancer cells, which confirmed an initial hypothesis^9–15^. Various mechanisms have been proposed in which salinomycin exerted its anti-cancer effects such as an autophagic cell death inducer^16^; signal transducer and activator of transcription 3 (STAT3), or Wnt signaling pathway inhibitor^17^; ATP-binding cassette (ABC) transporter inhibitor^16,18^; potent mitochondrial function inhibitor^16,19–22^. Side effects of salinomycin reported in clinical studies include tachycardia and mild tremor; however, none of the severe side effects such as alopecia, nausea, myelodepression, or gastrointestinal distress characteristic of traditional chemotherapeutic drugs, has been documented^23^.

Dichloroacetate (DCA) is a small synthetic molecule that is known as a pyruvate dehydrogenase kinase inhibitor. Its anticancer properties involve reversing the Warburg effect by switching ATP production back to oxidative phosphorylation^24–28^; reduction of mitochondrial membrane potential (ΨIM), and activation of mitochondrial potassium channels, which subsequently contribute to the induction of apoptosis in various cancers through the release of proapoptotic molecules such as cytochrome c (cyt c) and apoptosis inducing factor (AIF)^29,30^. Several features of DCA make it an attractive candidate for cancer therapy: it has a minimal effect on healthy cells^31^, good bioavailability^27^, and is a low cost drug. Additionally, DCA has been used to treat patients with congenital lactic acidosis in clinic settings for more than 40 years, hence its side effects are already well studied^32^. In the last decade, a number of articles have been published in favor of DCA, and it was proposed as an effective drug to treat neuroblastoma, breast, colon, lung, prostate, and other cancers^24,25,30,33^. A successful 1 phase clinical trial to treat patients with recurrent malignant brain tumors was completed in 2014 and it concluded DCA as safe, tolerable, and feasible for chronic administration^34^. Another 1 phase clinical trial performed with DCA on various advanced solid tumors supports these data^35^. Side effects caused by DCA can be categorized in two groups: neurological such as peripheral neuropathy, sedation, mood fluctuations, or disorientation and gastrointestinal such as heartburn, nausea, vomiting, or indigestion^36^.

A great number of scientific reports have shown that the multi-drug resistance phenotype in tumors correlates with the increased expression of particular ABC transporters, so-called multidrug resistance proteins (MRPs). P-glycoprotein (P-gp) was the first identified ABC transporter and it is thought to be responsible for multi-drug resistance in majority types of cancer. Some papers have suggested salinomycin as a possible P-gp inhibitor^18^, while DCA so far has never been identified to possess such characteristics. On the other hand, a recent study performed *in vivo* on mice with glioblastoma tumors has proposed that DCA could be used for prompt intracellular acidification that, in turn, may enhance the effectiveness of chemotherapeutic treatment^37–39^.

In the present study, we found that in the 2D and 3D cultures of HCT116 and DLD-1 cells, salinomycin and DCA exerted a synergistic cytotoxic effect, and provided experimental evidence that the mechanism of their synergism was related to DCA-induced inhibition of MRPs.

## Results

### DCA in combination with salinomycin synergistically inhibits the viability of HCT116 and DLD-1 cells in 2D culture

**Figure 1:**
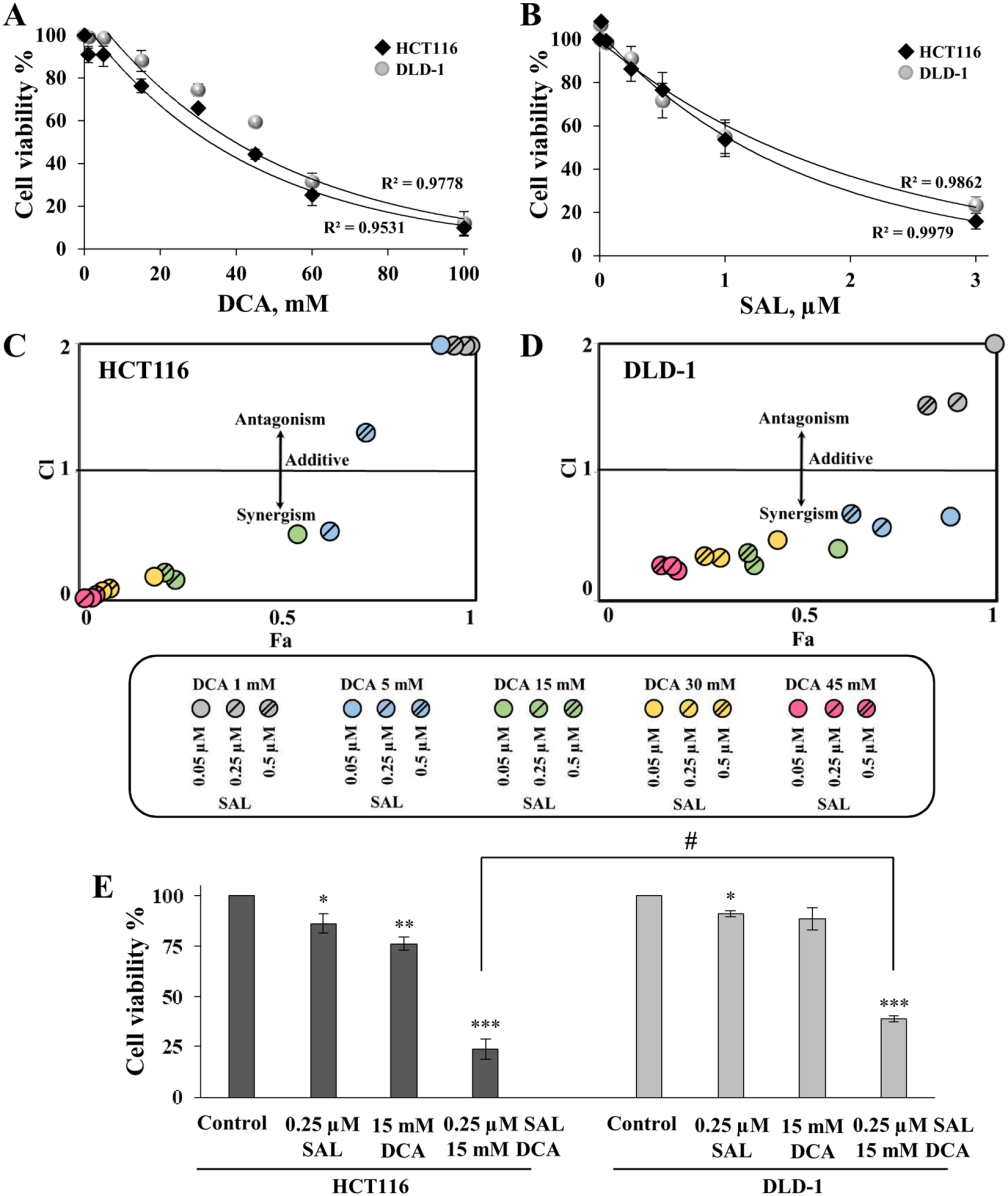
Cytotoxic effects of DCA and salinomycin in monotherapy and in combination on colorectal cancer cells in the 2D culture determined by the MTT assay. (**A**,**B**) A dose-response curve of cytotoxic effect of DCA (**A**) and salinomycin (**B**) alone on DLD-1 and HCT116 cell viability after 48-h treatment. **(C**,**D**) Fa-CI plot analysis of combination treatment of DCA and salinomycin (SAL) on DLD-1 (**C**) and HCT116 (**D**) cell viability. For visual purpose, all CI values above 2 were presented as equal to 2. (**E**) Effects of 0.25-μM salinomycin and 15-mM DCA and their combination on HCT116 and DLD-1 cell viability after 48-h treatment, determined by the MTT assay. Data are expressed as mean ± SEM calculated from 3 independent experiments (n = 3) measuring cell viability in 6 wells for each condition. *p < 0.05; **p < 0.01; ***p < 0.001 (compared to control); ^#^p < 0.05 (compared between groups).

Our first objective was to examine the effects of DCA and salinomycin in HCT116 and DLD-1 colorectal cancer cell 2D cultures by applying the compounds in monotherapy as well as in combination. The experimental design was made in accordance with the Chou-Talalay method for drug combination and their synergy quantification^40^. Initially, we performed dose response experiments with each drug alone and calculated IC_50_ values, which were 33.7 and 44.4 mM for DCA as well as 1.6 and 1.3 µM for salinomycin in HCT116 and DLD-1 cell lines, respectively (Fig. 1A,B). Doses for combination treatment were selected based on their cytotoxic effects in monotherapy, and only concentrations above IC_50_ were included in the study.

Next, we tested these doses on both cell lines in the non-constant ratio drug model study. Combination treatment significantly inhibited the proliferation of both cell lines in a dose-dependent manner (Fig. 1C,D and Supplementary Table S1). When DCA concentrations between 1 and 5 mM were combined with any tested salinomycin doses (0.05, 0.25, and 0.5 µM), only additive or antagonistic effects were observed in HCT116 cells; however, with every concentration of DCA above 15 mM, a strong synergistic response was achieved. In contrast, 5 mM or higher concentrations of DCA combined with any tested doses of salinomycin caused a synergistic cytotoxic effect in DLD-1 cells. As illustrated in Fig. 1E, when 0.25 µM of salinomycin was applied in monotherapy, cell viability decreased only to 86.1% ± 4.6% and 91.3% ± 1.5% in HCT116 and DLD-1 cells, respectively. A similar percentage in viability reduction was observed when cells were treated with 15-mM DCA alone: to 76.3% ± 3.2% and 88.6% ± 5.5% in HCT116 and DLD-1 cells, respectively. However, once the combination therapy was applied, cell viability decreased dramatically: to 23.7% ± 4.9% and 38.7% ± 1.3% for HCT116 and DLD-1 cells, respectively. There was no statistically significant difference in the cytotoxic effect between the cell lines when DCA and salinomycin were used in monotherapy; however, the effect of combination therapy was significantly stronger in the HCT116 than DLD-1 cell line (p < 0.05). A similar tendency of cell response to drugs was also observed with other concentrations of combination therapy, which contained DCA of 15 mM or higher.

**Figure 2:**
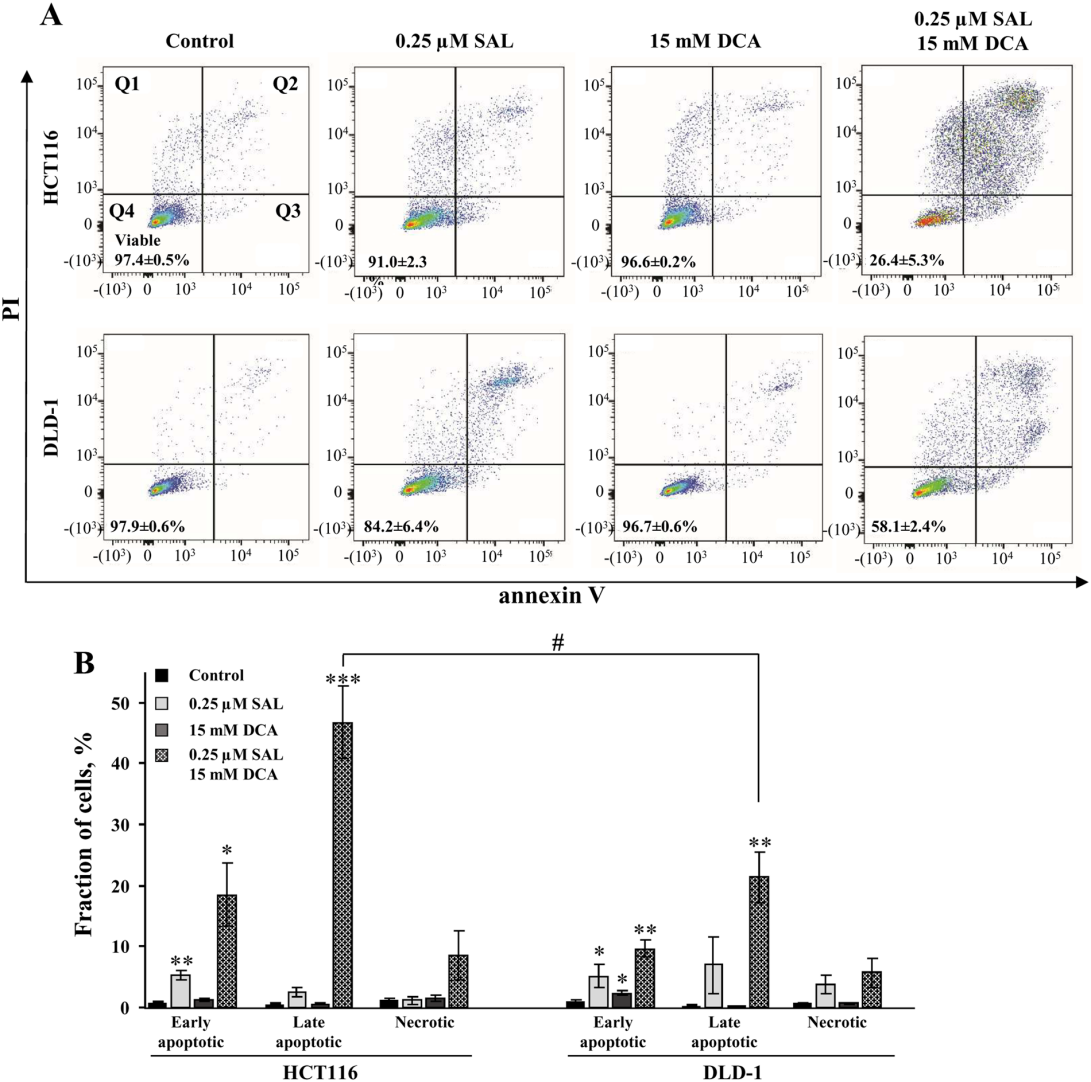
Flow cytometry analysis of annexin V- and PI-stained HCT116 and DLD-1 cells, undergoing treatment with salinomycin, DCA and their combination. (**A**) Dot plots represent responses to therapy with indicated compound(s) for HCT116 cells and DLD-1 cells. Q1 (necrosis) shows cells negative for annexin V labeling, but positive for PI staining. Q2 (late apoptosis) shows cells positive for annexin V labeling and positive for PI staining. Q3 (early apoptosis) shows cells positive for annexin V labeling, but negative for PI staining. Q4 (viable cells) shows cells negative for both annexin V labeling and PI staining. (**B**) Effects of 0.25-μM salinomycin and 15-mM DCA and their combination on HCT116 and DLD-1 cell viability after 48-h treatment, determined by flow cytometry. Data are expressed as mean ± SEM, averaged from 3 independent experiments. *p < 0.05; **p < 0.01; ***p < 0.001 (compared to control); ^#^p < 0.05 (compared between groups).

Similar effects of the used compounds on cell viability were obtained by flow cytometry analysis. Representative dot plots are shown in Fig. 2A, and the number of viable cells averaged from 3 independent experiments is indicated in each quadrant 4 (Q4). Also, the combination of salinomycin (0.25 µM) and DCA (15 mM) produced a synergistic effect dramatically increasing the early and late apoptotic cell populations after 48-h exposure in both HCT116 and DLD-1 cell lines compared with control and compared to the single agents (Fig. 2B). In addition, HCT116 cells were more sensitive to combination therapy than DLD-1 cells (p < 0.05).

### DCA and salinomycin cause a strong cytotoxic effect on colorectal cancer cell lines in the 3D cell culture

**Figure 3:**
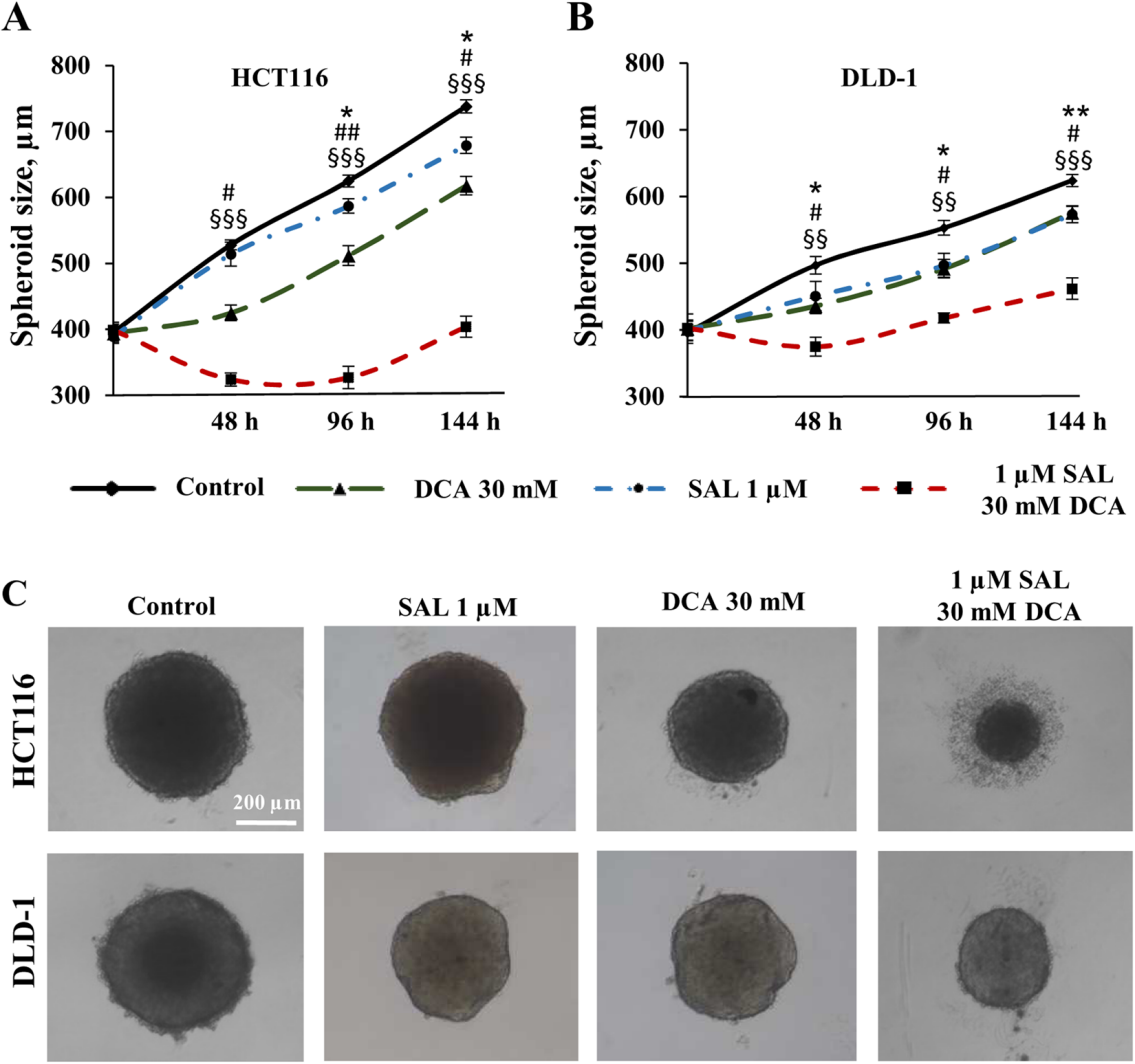
Cytotoxic effects of DCA, salinomycin, and their combination on the 3D colorectal cancer cell culture. The effect of 1 μM salinomycin, 30 mM DCA and their combination on HCT116 (**A**) and DLD-1 (**B**) multicellular spheroid size after 48, 96 and 144 h of treatment. Data are expressed as mean ± SEM calculated from 3 independent experiments (n = 3) measuring size of 6 spheroids for each condition. (**C**) Typical images of HCT116 and DLD-1 multicellular spheroids after 48 h of control or treatment with 1-μM salinomycin, 30-mM DCA, and their combination. *p < 0.05, **p < 0.01 (SAL compared to control); ^#^p < 0.05, ^##^p < 0.01 (DCA compared to control); ^§§^p < 0.01, ^§§§^p < 0.001 (SAL and DCA combination compared to control).

In order to determine whether combination therapy maintains its cytotoxic effect in the 3D cell culture, we applied the multicellular spheroid technique. An initial spheroid size evaluated after 48 h of cell seeding was 395 ± 17 µm (n = 216). The same day, selected spheroids were treated in monotherapy with 0.01, 0.25, 0.5, 1, and 5 µM of salinomycin, and 1, 10, 15, 30, 45, and 60 mM of DCA. Treatment efficacy was measured after 48, 96, and 144 h. Cytotoxic effects were assessed based on the multicellular spheroid size using SpheroidSizer V.1.0 software. As expected, the spheroids of both cell lines were less sensitive to drug doses that were effective in the 2D cell culture. The following doses were selected for combination therapy: 1 µM of salinomycin and 30 mM of DCA. Treatment efficacy was measured at the 3 different time points (48, 96, and 144 h). After 48 h, HCT116 cell spheroid size was unchanged after salinomycin, 20% (p < 0.05) smaller after DCA, and 39% (p < 0.001) smaller after combination treatment compared to control. At all examined time points, the effect of combination treatment was ~2-fold more effective than the sum of DCA and salinomycin monotherapies (Fig. 3A,C). In contrast, after 48 h, DLD-1 cell spheroid size was 10% (p < 0.05) smaller after salinomycin, 12% (p < 0.05) smaller after DCA and 25% (p < 0.01) smaller after combination treatment compared to control (Fig. 3B,C). The same tendency was observed after 96 and 144 h. Interestingly, DLD-1 cell spheroids were sensitive to salinomycin monotherapy at all time points, whereas in HCT116 cell spheroids, a significant effect was obtained only after 96 h.

### Expression of stem cell markers is up-regulated in the multicellular spheroid culture of DLD-1 cells

Our next objective was to investigate the rationale behind the altered chemotherapeutic sensitivity in the DLD-1 cell line, when grown in the 3D structure. We observed that in 3D culture DLD-1 cells were more sensitive to the monotherapy of salinomycin compared to HCT116 cells. Since salinomycin selectively targets cancer stem cells, we tested whether switching from 2D to 3D environment could have caused specific gene activation. We performed analysis of cancer cell stemness markers (*ALDH1A1*, *CEACAM5*, *ALCAM*, *LGR5*, *DPP4*, *CD133*, *CD24*, *CD29*, and *CD44*), epithelial-mesenchymal transition (EMT) markers (*SNAIL1*, *SNAIL2*, *CDH1*, and *CDH2*) and multipotency markers (*NANOG* and *POU5F1*) in HCT116 and DLD-1 cell 2D and 3D cultures in the absence of treatment. As presented in Table 1, no change (FC < 1.5 or p > 0.05) was observed in the regulation of multipotency genes in both cell lines, and only one marker (*SNAIL2*) was up-regulated among EMT markers in the DLD-1 cell line. In contrast, majority of the cell stemness markers (*CEACAM5*, *ALDH1A1*, *CD24*, *CD44*, and *CD133*) were significantly overexpressed in the DLD-1 cell line in a 3D environment, whereas only one stem cell marker (*DPP4*) was increased in the HCT116 cell line. These results suggest that higher potency of salinomycin monotherapy in DLD-1 cell 3D culture was achieved due to up-regulation of stemness genes.

**Table 1:** Gene expression analysis.

Gene Expression Analysis				
Genes	Cell lines and cultures			
	HCT116 3D/2D		DLD-1 3D/2D	
	FC	P value	FC	P value
EMT markers				
SNAIL1	−1.1	0.1821	−1.1	0.3618
SNAIL2	−1.4	0.0513	1.1	0.0429
CDH1	1.0	0.7252	1.0	0.5211
CDH2	1.1	0.3529	1.3	0.0821
Multipotency markers				
NANOG	1.0	0.9418	1.3	0.0200
POU5F1	1.2	0.1071	−1.1	0.4263
Cell stemness markers				
ALCAM	−1.2	0.0236	1.2	0.1517
CEACAM5	−1.1	0.9179	**24.3**	**0.0000**
ALDH1A1	0.0	>0.99	**8.5**	**0.0193**
LGR5	2.2	0.2784	**−1.9**	**0.0001**
DPP4	**1.7**	**0.0004**	**−1.5**	**0.0001**
CD24	1.0	0.6359	**2.0**	**0.0001**
CD29	−1.3	0.0055	−1.0	0.4036
CD44	−1.2	0.0661	**3.4**	**0.0001**
CD133	1.1	0.1786	**1.7**	**0.0001**

Relative expression of EMT, multipotency and cell stemness-related genes in DLD-1 and HCT116 2D and 3D cell cultures estimated by means of RT-qPCR (all experiments were repeated in independent biological triplicates).

### Putative mechanisms of action

#### A role of multidrug resistance proteins

**Figure 4:**
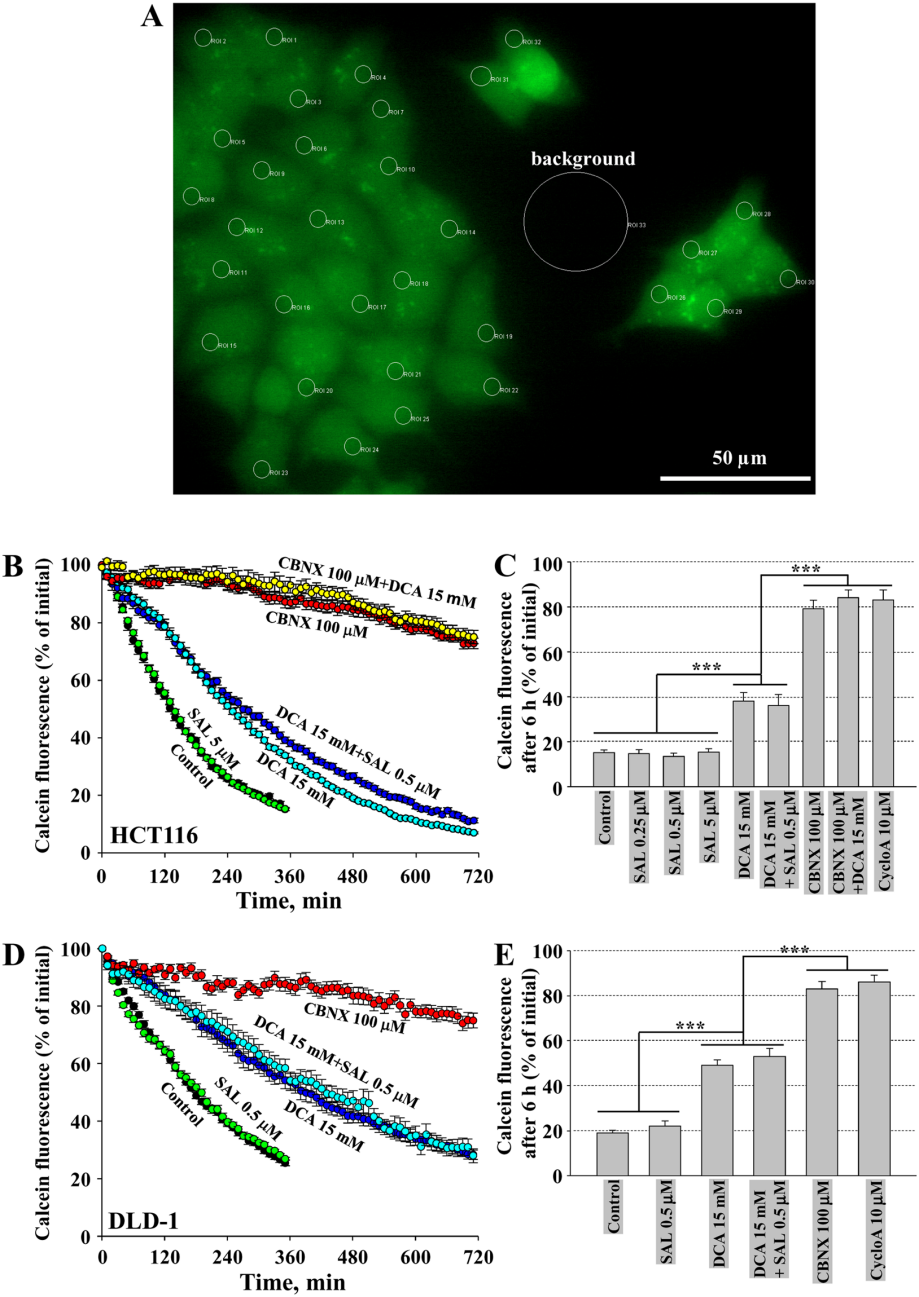
DCA inhibits MRP activity in HCT116 and DLD-1 cells. (**A**) View of calcein-AM loaded HCT116 cell group with regions of interest (ROIs) on every cell and ROI for background subtraction. (**B**) Typical calcein fluorescence decay in HCT116 cells under control (n = 38) and in the presence of salinomycin (5 µM; n = 32), DCA (15 mM; n = 27), DCA together with salinomycin (n = 32), CBNX (100 µM; n = 33), CBNX together with DCA (n = 35). (**C**) Calcein fluorescence in HCT116 cells after 6 h of recording relatively to initial fluorescence intensity under control and in the presence of indicated compounds. Data are expressed as mean ± SEM, averaged from 3 independent experiments. (**D**) Typical calcein fluorescence decay in DLD-1 cells under control (n = 37) and in the presence of salinomycin (0.5 µM; n = 16), DCA (15 mM; n = 17), DCA together with salinomycin (n = 21), CBNX (100 µM; n = 17). (**E**) Calcein fluorescence in DLD-1 cells after 6 h of recording relatively to initial fluorescence intensity under control and in the presence of indicated compounds. Data are expressed as mean ± SEM, averaged from 3 independent experiments. ***p < 0.001.

To examine a possibility that a synergistic effect of salinomycin and DCA was achieved due to modulation of MRP activity, we used the calcein assay (see Methods). To examine the constitutive activity of MRPs, we used cyclosporine A (10 µM) and carbenoxolone (CBNX, 100 µM). CBNX is a widely used inhibitor of connexin and pannexin hemichannels that also was shown to inhibit the activity of ABC transporters such as P-glycoprotein with IC_50_ of 81 µM^41^. Theoretically, the leakage of calcein from cells also may happen through connexin hemichannels; however, in our experiments, the involvement of hemichannels is hardly possible since they open only at positive membrane potentials^42^, while in HCT116 and DLD-1 cells using a whole-cell patch-clamp technique, we observed only negative potentials of several mV (not shown). As shown in Fig. 4, under control conditions, calcein fluorescence rapidly decayed; however, in the presence of cyclosporine A or CBNX, it remained relatively stable for more than 12 h. This simple test confirmed that MRPs in both cell types were extremely active, and the inhibition of MRP activity might enhance salinomycin effect on cancer cell viability.

Other authors have demonstrated that salinomycin reduced the expression of ABC transporters^16,18^ and removal of doxorubicin from cancer cells^43^. However, as can be seen in Figs. 4B–E, none of used concentrations of salinomycin had any effect on calcein removal from HCT116 and DLD-1 cells. Even longer preincubation (12 or 48 h) with salinomycin (0.5 µM) did not change calcein test results (not shown). In contrast, quite surprisingly for us, calcein fluorescence decay was significantly slowed-down by DCA. To our knowledge, this phenomenon has not been reported before. Thus, one of possible mechanisms by which the synergistic effect of salinomycin is achieved could be attributed to DCA-inhibited removal of salinomycin from cancer cells.

#### Intracellular pH

It is known that glycolytic nature of malignant tumors contributes to high levels of extracellular acidity in the tumor microenvironment (pH_o_ below 7), while pH_i_ is slightly alkaline (7.5–7.7) creating high proton gradients and oscillations that may contribute to cancer cell viability, proliferation, and invasion and response of tumors to various treatments such as chemotherapy, radiotherapy, and hyperthermia^44–50^.

Moreover, an uptake of drugs at acidic pH_o_ is impaired^51^. For example, the acidic pH_o_ increases the cellular uptake of weakly acidic drugs such as cyclophosphamide and cisplatin and retards the uptake of weakly basic drugs such as doxorubicin and vinblastine. We examined whether salinomycin, presumably acting on the activity of pH-regulating proteins, such as carbonic anhydrases, Na^+^/H^+^ exchanger, Na^+^/NCO_3_^−^ co-transporter, monocarboxylate transporter^52^, is capable of modifying pH_i_. Moreover, in contrast to the above-mentioned necessity of SLC5A8 transporter for delivery of DCA into the cells, there is a possibility that despite its ionization, it can pass through the plasma membrane. For instance, acetate (ACE) is widely used for experimental acidification of intracellular milieu, as we also did in our earlier studies^53^, hence we hypothesize that DCA may pass through the plasma membrane in the same manner and modify pH_i_ like ACE. Used concentrations of salinomycin and DCA had no effect on pH_o_.

**Figure 5:**
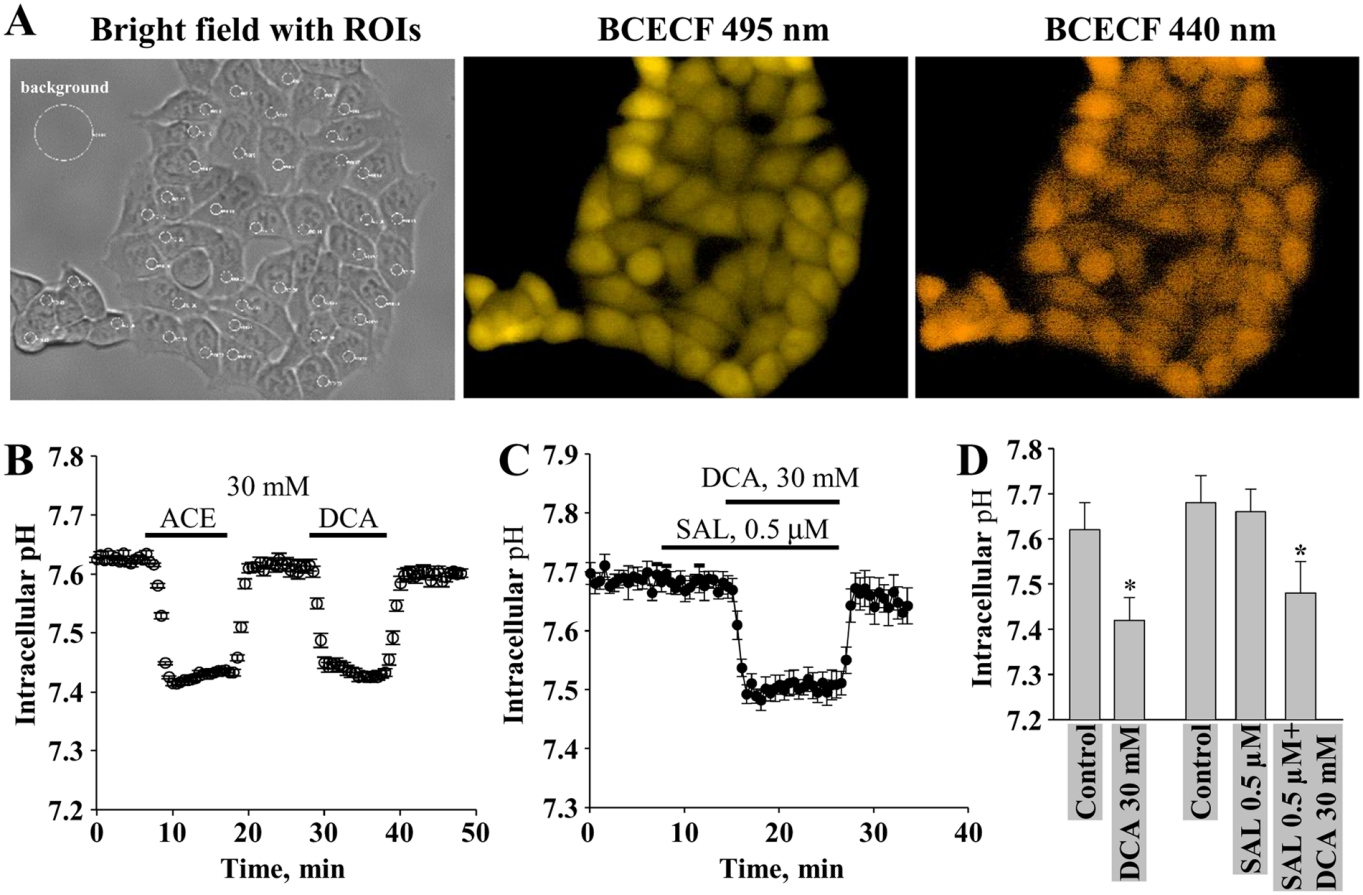
The effect of salinomycin and DCA on pH_i_ in HCT116 cells. (**A**) Bright field and BCECF fluorescence images at 495 nm and 440 nm excitation waves. (**B**) The effect of ACE (30 mM) and DCA (30 mM) alone on pH_i_ (n = 41). (**C**) The effect of salinomycin (0.5 µM) and DCA (30 mM) on pH_i_ (n = 24). (**D**) Summary of the effects of salinomycin and DCA on pH_i_. Horizontal bars in B and C indicate times of exposure to indicated compounds. Data in D are expressed as mean ± SEM, averaged from 3 independent experiments. *p < 0.05.

To measure pH_i_, we loaded cells with BCECF, a pH-sensitive fluorescent dye (see Methods). Under control conditions, pH_i_ in HCT116 cells was elevated and varied between 7.6 and 7.7. As shown in Fig. 5, salinomycin had no effect on pH_i_. In contrast, DCA (30 mM) alone or in the presence of salinomycin decreased pH_i_ by ~0.2 unit in HCT116 cells. Identical effects were documented in DLD-1 cells (data not shown).

In order to investigate whether pH_i_ modulation contributed to a synergistic cytotoxic effect, we performed a series of experiments in 2D and 3D cell cultures where DCA was replaced with ACE that does not affect the activity of PDK^28^ and, to our knowledge, the activity of MRP. As shown in Supplementary Fig. S1, no synergistic effect was observed in combination therapy with salinomycin and ACE in 2D and 3D culture of both cell lines, despite the fact that ACE decreased pH_i_ to the similar extent as did DCA. Thus, pH_i_ does not appear to be contributing to the synergistic effect.

## Discussion

In the present study, we have discovered the synergistic cytotoxic effect of two drugs – salinomycin and DCA – in HCT116 and DLD-1 colorectal cancer cell lines and proposed explanation of the mechanism of its action, which involves previously undisclosed activity of DCA.

Salinomycin has been recently re-discovered for its cytotoxic properties. While the first pilot therapeutic use of salinomycin in humans demonstrated promising results, it seemed that monotherapy with salinomycin was not sufficient to effectively eradicate the entire tumor, and many sequential studies have suggested that salinomycin can exert a much higher effect at lower doses when cells are previously sensitized by other chemotherapeutic drugs^54–57^.

DCA is a well-known drug that has been used in clinical settings for many years. Rapid absorption of DCA after oral administration was revealed in clinical studies^34,58^ or fast mechanism of action was determined by increased oxygen consumption rate *in vitro* after DCA application^59^. Even though some authors claim that DCA cell permeability depends on the expression of SLC5A8 transporter in the cells^60^, our measurements of DCA effects on pH_i_ (Fig. 5) suggest that DCA uptake is fast, even though SLC5A8 in HCT116 cells is absent^61^.

An accumulating body of evidence suggests that therapies having multiple targets result in greater benefits than single-targeted therapies. There are several studies suggesting a synergistic action of DCA or salinomycin with other compounds. Salinomycin combined with orally active epidermal growth factor receptor inhibitor gefitinib synergized to induce apoptosis via mitochondrial-lysosomal cross-talk in colorectal cancer cells^62^. Chloroquine in liver cancer cells with higher basal autophagy flux increased cytotoxic effect of salinomycin^63^. Salinomycin potentiated the effect of traditional chemotherapeutic drug 5-fluorouracil, the clinical use of which is limited due to rapid development of acquired resistance, by down-regulating cancer stem cells in hepatocellular carcinoma^64^. Similar results were obtained in colorectal cancer cells using 5-fluorouracil with DCA. This combination induced apoptosis through a caspase-dependent mitochondria pathway^65^. Salinomycin combined with natural polyphenol resveratrol induced apoptosis in human breast cancer cells by reactive oxygen species-mediated caspase activation^66^. Even though DCA-mediated protective autophagy and metformin-induced lactate accumulation limit their tumor-killing potential, their combination synergistically suppressed the growth of ovarian cancer cells^67^. Omeprazole, a proton pump inhibitor, in combination with DCA was more effective than either drug alone against fibrosarcoma and colon cancer cells as well as in animal experiments^68^. Taxol is an anti-mitotic agent that promotes apoptosis in cancer cells; however, the majority of patients develop resistance to it. Taxol resistance in oral cancer cells cultured under hypoxic conditions could be overcome by DCA^69^. Similarly, part of patients is not sensitive to cisplatin treatment. DCA could remarkably increase cisplatin-induced cell death in prostate cancer by promoting reactive oxygen species production^70^. Estradiol analogue with improved antimitotic activity in combination with DCA synergized against malignant breast carcinoma cells MCF-7^71^.

It is important that mechanisms of salinomycin and DCA action are not overlapping, which is a desirable characteristic of synergistic combination therapy. We have first found that the combination of these two drugs had a much stronger dose-dependent effect on HCT116 and DLD-1 colorectal cancer cells in the 2D culture compared with the effect observed in monotherapy experiments. Chou-Talalay analysis revealed that drugs acted synergistically (Fig. 1). These results motivated us to repeat combination therapy in the 3D cell culture. According to literature, most therapeutic approaches were less effective in 3D than 2D cultures^72^. Our results showed that combination therapy maintained a strong cytotoxic effect in the 3D cell culture and was significantly stronger than summary of both monotherapies (Fig. 3). We also observed that the HCT116 cell line was more sensitive to combination therapy than the DLD-1 cell line. To our surprise, the DLD-1 cell line had a higher sensitivity to monotherapy with salinomycin in the 3D environment compared with HCT116 (Fig. 3A,B). The 3D microenvironment recapitulates cell diversity that is observed *in vivo*: 3D systems may exhibit heterogenic phenotypes such as rapidly proliferating cells *versus* quiescent cancer stem cells. As mentioned previously, salinomycin is assumed to act preferably on cancer stem cells. Therefore, it may have a greater cytotoxic effect on the cancer cell population enriched with cancer stem cells. RT-qPCR analysis revealed that a number of cancer stem cell genes were significantly upregulated in the DLD-1 cell 3D culture compared with the 2D culture, whereas HCT116 cells exhibited only one significantly upregulated gene in the 3D cell culture (Table 1). This fact suggests that increased potency of salinomycin in the DLD-1 cell 3D culture could be attributed to augmented stemness of these cells.

Next, we investigated the possible mechanism of synergistic cytotoxic action of salinomycin and DCA. First, we examined the effects of both drugs on MRP activity. Using the calcein assay, we found that DCA, but not salinomycin, was responsible for delayed decay in calcein fluorescence, implying that a cytotoxic effect of salinomycin could be achieved due to DCA-induced intracellular retention of salinomycin (Fig. 4). To our knowledge, such mechanism of synergism has been never proposed before and not only it provides a possible explanation of our combination therapy, but also suggests that other chemotherapeutic drugs could be potentiated in a similar manner when used in combination with DCA. Subsequently, we explored the effects of salinomycin and DCA on pH_i_. Cells under alkaline pH_i_ were observed to proliferate, enter the cell cycle, differentiate, migrate, reduce apoptosis, and undergo malignant transformation – events that are critical in cancer formation and metastasis^73–75^. A recent *in vivo* study, performed on mice bearing glioblastoma tumors, has demonstrated that a single dose of DCA can be used as a pharmacological challenge to prompt tumor intracellular acidification; however, the mechanism of action was not fully disclosed^37^. Our *in vitro* results supported this finding and showed that while salinomycin had no effect on pH_i_, DCA like ACE, which is often used to acidify an intracellular milieu under experimental conditions^53^, reduced pH_i_ by ~0.2 (Fig. 5), which could contribute to increased cell sensitivity to combination therapy and apoptotic signaling. However, our experiments replacing DCA by ACE show that reduced pH_i_ is not a sufficient factor for potentiation of salinomycin effect (Supplementary Fig. S1).

In summary, our findings indicate that the combination therapy of salinomycin and DCA could be an effective option for colorectal cancer treatment and provide the first mechanistic explanation of the synergistic action of these compounds. Our further studies are focused on *in vivo* testing of salinomycin and DCA combination using an induced colorectal cancer model in mice.

## Materials and Methods

### Cell lines and reagents

Human colorectal carcinoma DLD-1 and HCT116 cell lines were obtained from the American Type Culture Collection (Rockville, Maryland, USA). Both cell lines were maintained in RPMI-1640 cell culture medium, which was supplemented with 10% fetal bovine serum (Gibco, Germany), 2 mM glutamine (Gibco, Germany), 1 mM sodium pyruvate (Gibco, Germany), 100 UI/mL penicillin (Sigma Aldrich, USA), and 0.1 mg/mL streptomycin (Sigma Aldrich, USA). Cell culturing was performed in 25-cm^2^ plastic cell culture flasks at 37 °C in a 5% CO_2_ humidified incubator. Salinomycin (Sigma Aldrich, USA) was prepared in stock by dissolving it in DMSO (96%) with the concentration of 5 mM and stored in aliquots at −20 °C. DCA and ACE (Sigma Aldrich, USA) were dissolved in PBS at a concentration of 300 mM and maintained at 4 °C. 3-(4,5-dimethylthiazol-2-yl)-2,5-diphenyltetrazolium bromide (MTT) powder (ThermoFisher Scientific, USA) was purchased from Sigma Aldrich, USA and prepared in 5 mg/mL stock and stored at 4 °C.

### Cell Viability Assay

To determine the effect of combination therapy on colorectal cancer cell proliferation, we employed an MTT assay. Cells were seeded in 96-well plates at the density of 15,000 (DLD-1) and 20,000 (HCT116) cells per well in 200 μL of RPMI-1640 culture media in order to reach 70–80% confluence. After 24-h incubation at 37 °C, cells were treated with salinomycin (0.01, 0.05, 0.25, 0.5, 1 μM), DCA (1, 5, 15, 30, 45 mM), or both (mixture of concentrations selected from monotherapy regimens of both agents, detailed in Table S1). Various concentrations of DMSO were added as a negative vehicle control for salinomycin and combination therapy. After treatment, cells were cultured for 48 h, and then the MTT reagent was added to each well and incubated for 1.5 h at 37 °C. Formazan crystals then were dissolved in DMSO, and the absorbance of each well was measured by a plate reader at a test wavelength of 490 nm. The absorbance values of each drug group was expressed as a percentage change of that of control group. A synergistic effect was evaluated based on the Chou-Talalay method. First, we estimated the intercept and the slope from a dose response curve and calculated a median effective dose (ED_50_). ED_50_ was used to evaluate the combination index (CI), which is recognized as an essential measure for the determination of combination effect. Subsequently, we employed Fa-CI plot (Fa – fraction affected) and isobologram analysis to determine whether the interactions between two compounds were additive, synergistic, or antagonistic. Both Fa-CI plot and isobologram yield identical conclusion of synergism or antagonism. The Fa-CI plot is effect-oriented while the isobologram is dose-oriented. However, we preferred to display our results in the Fa-CI plot as it is visually more convenient than the isobologram since data points in the isobologram usually overlap. Data points on the Fa-CI plot were based on each dose of combination therapy. According to the Chou-Talalay method, all dots plotted under the line indicated synergism (CI < 1), dots falling on the line represented additive interactions (CI = 1), and the dots located above the line showed antagonism (CI > 1).

### 3D Cell Culture Experimental Design

Multicellular tumor spheroids (MTSs) were formed as described previously^76^ with slight alterations to the protocol. Briefly, in order to reach a standard spheroid size of ~400 µm after 48-h seeding, cells were placed in a 1% agarose pre-coated 96-well U-bottomed plate at a cell density of 1300 and 4500 cells per well for HCT116 and DLD-1 cell lines, respectively, and centrifuged at 1000 g for 10 min. After 48 h, spheroids were treated by applying monotherapy and combination therapy. Pictures of cells were taken with an inverted optical microscope Eclipse TS100 and a digital camera DS-Fi2 (Nikon, Japan) every other day for 8 days. Spheroid dimensions were assessed by SpheroidSizer 1.0^77^.

### Cell cycle and apoptosis assessment by flow cytometry and annexin V/propidium iodide double-staining

Cells were seeded in a 6-well plate at a density of 300,000 (HCT116) and 400,000 (DLD-1) cells per well. After 24 h, cells were treated with salinomycin, DCA, or drug combination at doses selected during the cell viability assay experiment: 0.25 μM of salinomycin, 15 mM of DCA, and the combination of two. After 48 h, cells were collected and washed by centrifugation. For apoptosis detection, we used an Annexin V-FITC apoptosis detection kit (Sigma Aldrich, USA). Cells were resuspended in 500 μL of binding buffer; subsequently, 5-μL annexin V conjugated with FITC and 5-μL propidium iodide (PI) solution (100 μg/mL) were added to the mixture, and cells were maintained at room temperature in the dark for 5 min. Cells were analyzed for apoptosis by a BD LSR II flow cytometer (BD Biosciences, USA) and an FACS Diva software package (BD Biosciences, USA).

### Measurement of intracellular pH

For pH_i_ measurements, cells grown onto glass coverslips were loaded with a cell-permeant form of 2′,7′-bis-(2-carboxyethyl)-5- (and -6)-carboxyfluorescein (BCECF-AM) (ThermoFisher Scientific, USA) by incubating cells in the Krebs–Ringer solution (in mM: NaCl, 140; KCl, 4; CaCl_2_, 2; MgCl_2_, 1; glucose, 5; pyruvate, 2; Hepes, 5 (pH 7.4)) containing 2 μM of BCECF-AM for 5 min. Then cells were washed with RPMI-1640 medium and transferred for fluorescence recording to the experimental chamber with the constant flow through perfusion system mounted on the stage of inverted microscope Olympus IX81 equipped with the Orca-R^2^ cooled digital camera, fluorescence excitation system MT10 (Olympus Life Science Europa Gmbh, Hamburg, Germany), and fluorescence imaging system XCELLENCE. Measurements pH_i_ were performed in control or indicated compound-containing RPMI-1640 medium. The dye was alternately excited with 440-nm and 495-nm light, and the emitted light was filtered at 540 nm and recorded. The emitted light from 495-nm excitation is pH sensitive, whereas that from 440-nm excitation is relatively pH insensitive. Thus, the ratio of emitted light at two excitation wavelengths (background subtracted) is a function of pH. Ratios were converted to pH_i_ values based on a calibration curve. The latter was obtained by using ionophore nigericin at a concentration of 20 µM in the presence of 140-mM potassium to equilibrate the intracellular pH with extracellular medium of different pH. All experiments were performed at room temperature. To minimize dye bleaching, imaging was performed in a time-lapse mode, i.e., cells were periodically exposed (every 30 s) to a low-intensity light for 100 ms.

### Calcein assay

Calcein is an excellent substrate of MRPs^78^. Therefore, we used a calcein assay to examine the effect of DCA; salinomycin; widely used ABC transporter (ATP-binding cassette transporter) inhibitor cyclosporine A (Sigma Aldrich, USA); and pannexin and connexin hemichannel inhibitor carbenoxolone (CBNX) (Sigma Aldrich, USA) that also was shown to be a P-gp inhibitor^41^, on calcein removal from HCT116 and DLD-1 cells. Cells were loaded with calcein-AM (Molecular Probes, USA) by incubating the cells in the RPMI-1640 solution containing 4 µM of calcein-AM for 30 min at 37 °C in a CO_2_ incubator. All compounds used in this assay were applied right before the beginning of time-lapse recording. The dye was excited with 490 nm light, and the emitted light was filtered at 540 nm and recorded. Cells were periodically exposed (every 10 min) to a low-intensity light for 100 ms. Time-lapse imaging of calcein fluorescence in the RPMI-1640 cell culture medium was performed at 37 °C in a humidified atmosphere of 5% CO_2_ using an incubation system INUBG2E-ONICS (Tokai Hit, Shizuoka-ken, Japan) with an incubator, mounted on the stage of the motorized Olympus IX81 microscope. Calcein fluorescence was measured in the regions of interest (ROIs) placed on every cell of the cell group. Due to cell motility, the positions of every ROI were corrected for every frame. Dispersion of calcein load in different cells was compensated by normalizing the initial fluorescence in every cell to 100%.

### RNA extraction

After 4 days, cells from both 2D and 3D cultures were collected. Total RNA was isolated from roughly 2 × 10^6^ cells using Quick RNA MiniPrep (Zymo Research, USA) following the instructions provided by the manufacturer. The quantity and quality of RNA were measured with a Nanodrop spectrophotometer at 260/280 nm (ThermoFisher Scientific, USA).

### RT-qPCR

A revert Aid RT Kit (ThermoFisher Scientific, USA) was employed in order to synthesize cDNA. As per manufacturer’s instructions, a total of 1 µg RNA was used for cDNA synthesis. Real-time qPCR was performed with Kapa SYBR Fast qPCR Master Mix (2X) (Kapa Biosystems, USA) using Realplex4 Mastercycler thermocycler (Eppendorf, USA) under the following conditions: 95 °C (3 min) for polymerase activation and 40 2-step cycles of 95 °C (3 s) and 60 °C (30 s) for denaturation and annealing/extension respectively. We used 10 µL solution for every reaction in a 96-well plate containing 1-µL cDNA, 5-µL Kapa SYBR FAST qPCR Master Mix, 2 forward and reverse primers, and nuclease-free water up to 10 µL. Expression of the housekeeping gene, HPRT1, was used as an endogenous control. qPCR products were analyzed in triplicates, and the fold change of the gene expression was quantified relative to the housekeeping gene, HPRT1, using the ΔΔCt method. All primers were purchased from Biolegio (Netherlands)^79^.

### Statistical analysis

All data were expressed as mean ± standard error mean (SEM) from at least three independent experiments. Statistical analysis was performed using Sigma Plot 10.0 software. Comparisons between two values were performed using the Student t test. Synergism of DCA and salinomycin was analyzed with the Fa-CI plot, and CI calculations were done according to the Chou-Talalay method using the CompuSyn 2.0 software (ComboSyn, Inc., Paramus, NJ, USA). CI values below 1 suggest synergy, whereas CI values above 1 indicate antagonism. For the comparison of gene expression profile between 2D and 3D cell cultures, a fold change (FC) value was calculated. Only gene expression with p < 0.05 and an absolute FC of ≥1.5 were considered as significant^80^.

## Electronic supplementary material

Supplementary Figure 1

## Data Availability

All data generated or analyzed during this study are included in this published article and its Supplementary Information file.

## References

[1] Marley AR, Nan H (2016). Epidemiology of colorectal cancer. Int J Mol Epidemiol Genet.

[2] Marshall JL (2007). Adjuvant Therapy for Stage II and III Colon Cancer: Consensus Report of the International Society of Gastrointestinal Oncology. Gastrointest Cancer Res.

[3] Hagan S, Orr MC, Doyle B (2016). Targeted therapies in colorectal cancer-an integrative view by PPPM. Epma J.

[4] Tournigand C (2004). FOLFIRI followed by FOLFOX6 or the reverse sequence in advanced colorectal cancer: a randomized GERCOR study. J Clin Oncol.

[5] Oliver Metzig M (2016). Inhibition of caspases primes colon cancer cells for 5-fluorouracil-induced TNF-alpha-dependent necroptosis driven by RIP1 kinase and NF-kappaB. Oncogene.

[6] Longley DB, Harkin DP, Johnston PG (2003). 5-fluorouracil: mechanisms of action and clinical strategies. Nat Rev Cancer.

[7] Bayat Mokhtari R (2017). Combination therapy in combating cancer. Oncotarget.

[8] Gupta PB (2009). Identification of selective inhibitors of cancer stem cells by high-throughput screening. Cell.

[9] Kim KY (2015). Salinomycin enhances doxorubicin-induced cytotoxicity in multidrug resistant MCF-7/MDR human breast cancer cells via decreased efflux of doxorubicin. Mol Med Rep.

[10] An H (2015). Salinomycin possesses anti-tumor activity and inhibits breast cancer stem-like cells via an apoptosis-independent pathway. Biochem Biophys Res Commun.

[11] Koo KH (2013). Salinomycin induces cell death via inactivation of Stat3 and downregulation of Skp2. Cell Death Dis.

[12] Zhou S (2014). Salinomycin Suppresses PDGFRbeta, MYC, and Notch Signaling in Human Medulloblastoma. Austin J Pharmacol Ther.

[13] Zhang Y (2017). Salinomycin Exerts Anticancer Effects on PC-3 Cells and PC-3-Derived Cancer Stem Cells *In Vitro* and *In Vivo*. Biomed Res Int.

[14] Lee HG (2017). Salinomycin reduces stemness and induces apoptosis on human ovarian cancer stem cell. J Gynecol Oncol.

[15] Oak Prajakta S., Kopp Florian, Thakur Chitra, Ellwart Joachim W., Rapp Ulf R., Ullrich Axel, Wagner Ernst, Knyazev Pjotr, Roidl Andreas (2012). Combinatorial treatment of mammospheres with trastuzumab and salinomycin efficiently targets HER2-positive cancer cells and cancer stem cells. International Journal of Cancer.

[16] Fuchs D, Daniel V, Sadeghi M, Opelz G, Naujokat C (2010). Salinomycin overcomes ABC transporter-mediated multidrug and apoptosis resistance in human leukemia stem cell-like KG-1a cells. Biochem Biophys Res Commun.

[17] Lu D (2011). Salinomycin inhibits Wnt signaling and selectively induces apoptosis in chronic lymphocytic leukemia cells. Proc Natl Acad Sci USA.

[18] Riccioni R (2010). The cancer stem cell selective inhibitor salinomycin is a p-glycoprotein inhibitor. Blood Cells Mol Dis.

[19] Mitani M, Yamanishi T, Miyazaki Y, Otake N (1976). Salinomycin effects on mitochondrial ion translocation and respiration. Antimicrob Agents Chemother.

[20] Pedersen SF, Stock C (2013). Ion channels and transporters in cancer: pathophysiology, regulation, and clinical potential. Cancer Res.

[21] Kim KY (2011). Salinomycin-induced apoptosis of human prostate cancer cells due to accumulated reactive oxygen species and mitochondrial membrane depolarization. Biochem Biophys Res Commun.

[22] Ketola K (2012). Salinomycin inhibits prostate cancer growth and migration via induction of oxidative stress. Br J Cancer.

[23] Naujokat, C. & Steinhart, R. Salinomycin as a drug for targeting human cancer stem cells. *J Biomed Biotechnol*, 950658 (2012). 10.1155/2012/950658 PMC3516046 23251084

[24] Wong JY, Huggins GS, Debidda M, Munshi NC, De Vivo I (2008). Dichloroacetate induces apoptosis in endometrial cancer cells. Gynecol Oncol.

[25] Cao W (2008). Dichloroacetate (DCA) sensitizes both wild-type and over expressing Bcl-2 prostate cancer cells *in vitro* to radiation. Prostate.

[26] Papandreou I, Goliasova T, Denko NC (2011). Anticancer drugs that target metabolism: Is dichloroacetate the new paradigm?. Int J Cancer.

[27] Michelakis ED, Webster L, Mackey JR (2008). Dichloroacetate (DCA) as a potential metabolic-targeting therapy for cancer. Br J Cancer.

[28] Stockwin LH (2010). Sodium dichloroacetate selectively targets cells with defects in the mitochondrial ETC. Int J Cancer.

[29] Sun RC, Board PG, Blackburn AC (2011). Targeting metabolism with arsenic trioxide and dichloroacetate in breast cancer cells. Mol Cancer.

[30] Bonnet S (2007). A mitochondria-K+ channel axis is suppressed in cancer and its normalization promotes apoptosis and inhibits cancer growth. Cancer Cell.

[31] Stacpoole PW (1989). The pharmacology of dichloroacetate. Metabolism.

[32] Stacpoole PW (2006). Controlled clinical trial of dichloroacetate for treatment of congenital lactic acidosis in children. Pediatrics.

[33] Sun RC (2010). Reversal of the glycolytic phenotype by dichloroacetate inhibits metastatic breast cancer cell growth *in vitro* and *in vivo*. Breast Cancer Res Treat.

[34] Dunbar EM (2014). Phase 1 trial of dichloroacetate (DCA) in adults with recurrent malignant brain tumors. Invest New Drugs.

[35] Chu QS (2015). A phase I open-labeled, single-arm, dose-escalation, study of dichloroacetate (DCA) in patients with advanced solid tumors. Invest New Drugs.

[36] Michelakis ED (2010). Metabolic modulation of glioblastoma with dichloroacetate. Sci Transl Med.

[37] Albatany M, Li A, Meakin S, Bartha R (2018). Dichloroacetate induced intracellular acidification in glioblastoma: *in vivo* detection using AACID-CEST MRI at 9.4 Tesla. J Neurooncol.

[38] Izumi H (2003). Cellular pH regulators: potentially promising molecular targets for cancer chemotherapy. Cancer Treat Rev.

[39] Neri D, Supuran CT (2011). Interfering with pH regulation in tumours as a therapeutic strategy. Nat Rev Drug Discov.

[40] Chou TC (2006). Theoretical basis, experimental design, and computerized simulation of synergism and antagonism in drug combination studies. Pharmacol Rev.

[41] Achilli TM, McCalla S, Meyer J, Tripathi A, Morgan JR (2014). Multilayer spheroids to quantify drug uptake and diffusion in 3D. Mol Pharm.

[42] Sáez Juan C., Retamal Mauricio A., Basilio Daniel, Bukauskas Feliksas F., Bennett Michael V.L. (2005). Connexin-based gap junction hemichannels: Gating mechanisms. Biochimica et Biophysica Acta (BBA) - Biomembranes.

[43] Kim Kwang-Youn, Park Kwang-Il, Kim Sang-Hun, Yu Sun-Nyoung, Park Sul-Gi, Kim Young, Seo Young-Kyo, Ma Jin-Yeul, Ahn Soon-Cheol (2017). Inhibition of Autophagy Promotes Salinomycin-Induced Apoptosis via Reactive Oxygen Species-Mediated PI3K/AKT/mTOR and ERK/p38 MAPK-Dependent Signaling in Human Prostate Cancer Cells. International Journal of Molecular Sciences.

[44] Fukumura D (2001). Hypoxia and acidosis independently up-regulate vascular endothelial growth factor transcription in brain tumors *in vivo*. Cancer Res.

[45] Lopez-Lazaro M (2006). HIF-1: hypoxia-inducible factor or dysoxia-inducible factor?. Faseb J.

[46] Gordan JD, Bertout JA, Hu CJ, Diehl JA, Simon MC (2007). HIF-2alpha promotes hypoxic cell proliferation by enhancing c-myc transcriptional activity. Cancer Cell.

[47] Masson N, Ratcliffe PJ (2014). Hypoxia signaling pathways in cancer metabolism: the importance of co-selecting interconnected physiological pathways. Cancer Metab.

[48] Xu L, Fidler IJ (2000). Acidic pH-induced elevation in interleukin 8 expression by human ovarian carcinoma cells. Cancer Res.

[49] Rofstad EK, Mathiesen B, Kindem K, Galappathi K (2006). Acidic extracellular pH promotes experimental metastasis of human melanoma cells in athymic nude mice. Cancer Res.

[50] Xu L, Fukumura D, Jain RK (2002). Acidic extracellular pH induces vascular endothelial growth factor (VEGF) in human glioblastoma cells via ERK1/2 MAPK signaling pathway: mechanism of low pH-induced VEGF. J Biol Chem.

[51] Wojtkowiak JW, Verduzco D, Schramm KJ, Gillies RJ (2011). Drug resistance and cellular adaptation to tumor acidic pH microenvironment. Mol Pharm.

[52] Parks SK, Pouyssegur J (2017). Targeting pH regulating proteins for cancer therapy-Progress and limitations. Semin Cancer Biol.

[53] Skeberdis VA, Rimkutė L, Skeberdytė A, Paulauskas N, Bukauskas FF (2011). pH-dependent modulation of connexin-based gap junctional uncouplers. J. Physiol..

[54] Zhou Y (2015). Salinomycin decreases doxorubicin resistance in hepatocellular carcinoma cells by inhibiting the beta-catenin/TCF complex association via FOXO3a activation. Oncotarget.

[55] Mirkheshti N (2016). Dual targeting of androgen receptor and mTORC1 by salinomycin in prostate cancer. Oncotarget.

[56] Booth L (2014). HDAC inhibitors enhance the lethality of low dose salinomycin in parental and stem-like GBM cells. Cancer Biol Ther.

[57] Xiao Z, Sperl B, Ullrich A, Knyazev P (2014). Metformin and salinomycin as the best combination for the eradication of NSCLC monolayer cells and their alveospheres (cancer stem cells) irrespective of EGFR, KRAS, EML4/ALK and LKB1 status. Oncotarget.

[58] Stacpoole PW, Henderson GN, Yan Z, James MO (1998). Clinical pharmacology and toxicology of dichloroacetate. Environ Health Perspect.

[59] Sutendra G (2013). Mitochondrial activation by inhibition of PDKII suppresses HIF1a signaling and angiogenesis in cancer. Oncogene.

[60] Babu E (2011). Role of SLC5A8, a plasma membrane transporter and a tumor suppressor, in the antitumor activity of dichloroacetate. Oncogene.

[61] Thangaraju Muthusamy, Cresci Gail, Itagaki Shiro, Mellinger John, Browning Darren D., Berger Franklin G., Prasad Puttur D., Ganapathy Vadivel (2008). Sodium-Coupled Transport of the Short Chain Fatty Acid Butyrate by SLC5A8 and Its Relevance to Colon Cancer. Journal of Gastrointestinal Surgery.

[62] Zou ZZ (2017). Synergistic induction of apoptosis by salinomycin and gefitinib through lysosomal and mitochondrial dependent pathway overcomes gefitinib resistance in colorectal cancer. Oncotarget.

[63] Xie F (2016). Codelivery of salinomycin and chloroquine by liposomes enables synergistic antitumor activity *in vitro*. Nanomedicine (Lond).

[64] Wang F (2014). The synergistic *in vitro* and *in vivo* antitumor effect of combination therapy with salinomycin and 5-fluorouracil against hepatocellular carcinoma. PloS one.

[65] Tong J (2011). Synergistic antitumor effect of dichloroacetate in combination with 5-fluorouracil in colorectal cancer. J Biomed Biotechnol.

[66] Dewangan J (2017). Novel combination of salinomycin and resveratrol synergistically enhances the anti-proliferative and pro-apoptotic effects on human breast cancer cells. Apoptosis.

[67] Li B (2016). Dichloroacetate and metformin synergistically suppress the growth of ovarian cancer cells. Oncotarget.

[68] Ishiguro T, Ishiguro M, Ishiguro R, Iwai S (2012). Cotreatment with dichloroacetate and omeprazole exhibits a synergistic antiproliferative effect on malignant tumors. Oncol Lett.

[69] Xie Q (2015). Combination of Taxol(R) and dichloroacetate results in synergistically inhibitory effects on Taxol-resistant oral cancer cells under hypoxia. Mol Med Rep.

[70] Zeng S, Liang H, Guan G (2016). Dichloroacetate enhances the cytotoxic effect of Cisplatin via decreasing the level of FOXM1 in prostate cancer. Int J Clin Med.

[71] Stander XX, Stander BA, Joubert AM (2015). Synergistic anticancer potential of dichloroacetate and estradiol analogue exerting their effect via ROS-JNK-Bcl-2-mediated signalling pathways. Cell Physiol Biochem.

[72] Hirschhaeuser F, Leidig T, Rodday B, Lindemann C, Mueller-Klieser W (2009). Test system for trifunctional antibodies in 3D MCTS culture. J Biomol Screen.

[73] Hulikova A, Aveyard N, Harris AL, Vaughan-Jones RD, Swietach P (2014). Intracellular carbonic anhydrase activity sensitizes cancer cell pH signaling to dynamic changes in CO2 partial pressure. J Biol Chem.

[74] Swietach P, Rossini A, Spitzer KW, Vaughan-Jones RD (2007). H+ ion activation and inactivation of the ventricular gap junction: a basis for spatial regulation of intracellular pH. Circ Res.

[75] McIntyre A (2016). Disrupting Hypoxia-Induced Bicarbonate Transport Acidifies Tumor Cells and Suppresses Tumor Growth. Cancer Res.

[76] Friedrich J, Seidel C, Ebner R, Kunz-Schughart LA (2009). Spheroid-based drug screen: considerations and practical approach. Nat Protoc.

[77] Chen, W. *et al*. High-throughput image analysis of tumor spheroids: a user-friendly software application to measure the size of spheroids automatically and accurately. *J Vis Exp* (2014). 10.3791/51639 PMC4212916 25046278

[78] Glavinas H, Krajcsi P, Cserepes J, Sarkadi B (2004). The role of ABC transporters in drug resistance, metabolism and toxicity. Curr Drug Deliv.

[79] Livak KJ, Schmittgen TD (2001). Analysis of relative gene expression data using real-time quantitative PCR and the 2(-Delta Delta C(T)) Method. Methods.

[80] McCarthy DJ, Smyth GK (2009). Testing significance relative to a fold-change threshold is a TREAT. Bioinformatics.

