# Supplementary material for: Dichloroacetate and Salinomycin Exert a Synergistic Cytotoxic Effect in Colorectal Cancer Cell Lines
Source: Sci Rep. 2018 Dec 10;8:17744. doi: 10.1038/s41598-018-35815-4 (PMC6288092; doi:10.1038/s41598-018-35815-4)
Supplement: Supplementary file 1 — Supplementary Figure 1 [file 41598_2018_35815_MOESM1_ESM.doc]

**Dichloroacetate and Salinomycin Exert a Synergistic Cytotoxic Effect in Colorectal Cancer Cell Lines**

Aistė Skeberdytė^1*^, Ieva Sarapinienė^2^, Jan Alexander-Krasko^3^, Vaidotas Stankevičius^3,4^, Kęstutis Sužiedėlis^1,3^, Sonata Jarmalaitė^1^

^1^ Institute of Biosciences, Life Sciences Center, Vilnius University, Saulėtekio al 7, LT-10222, Vilnius, Lithuania.

^2^ Institute of Cardiology, Lithuanian University of Health Sciences, Sukilėlių pr 15, LT-50162, Kaunas, Lithuania

^3^ National Cancer Institute, Santariškių gv 1, LT-08660, Vilnius, Lithuania.

^4^ Institute of Biotechnology, Life Sciences Center, Vilnius University, Saulėtekio al 7, LT-10222, Vilnius, Lithuania.

**Supplementary Table 1. Concentration-dependent combined effects of salinomycin and DCA in HCT116 and DLD-1 cell lines.** Fa, fraction affected; CI, combination index. CI of < 1 represents synergism, CI of 1 or close to 1 represents additive effects, and CI of > 1 represents antagonism.

| **Dose DCA (mM)** | **Dose SAL (μM)** | **HCT116** | | **DLD-1** | |
| --- | --- | --- | --- | --- | --- |
|  |  | **Fa** | **CI** | **Fa** | **CI** |
| 1 | 0.05 | 0.99 | 6.632 | 0.99 | 3.850 |
| 5 | 0.05 | 0.94 | 2.981 | 0.88 | 0.649 |
| 15 | 0.05 | 0.55 | 0.534 | 0.6 | 0.409 |
| 30 | 0.05 | 0.19 | 0.178 | 0.45 | 0.477 |
| 45 | 0.05 | 0.03 | 0.031 | 0.19 | 0.264 |
| 1 | 0.25 | 0.99 | 33.158 | 0.9 | 1.543 |
| 5 | 0.25 | 0.63 | 0.554 | 0.71 | 0.567 |
| 15 | 0.25 | 0.24 | 0.180 | 0.39 | 0.288 |
| 30 | 0.25 | 0.06 | 0.056 | 0.3 | 0.341 |
| 45 | 0.25 | 0.01 | 0.011 | 0.18 | 0.278 |
| 1 | 0.5 | 0.99 | 35.167 | 0.82 | 1.515 |
| 5 | 0.5 | 0.72 | 1.316 | 0.63 | 0.675 |
| 15 | 0.5 | 0.22 | 0.218 | 0.37 | 0.360 |
| 30 | 0.5 | 0.07 | 0.083 | 0.26 | 0.345 |
| 45 | 0.5 | 0.03 | 0.043 | 0.16 | 0.278 |

**
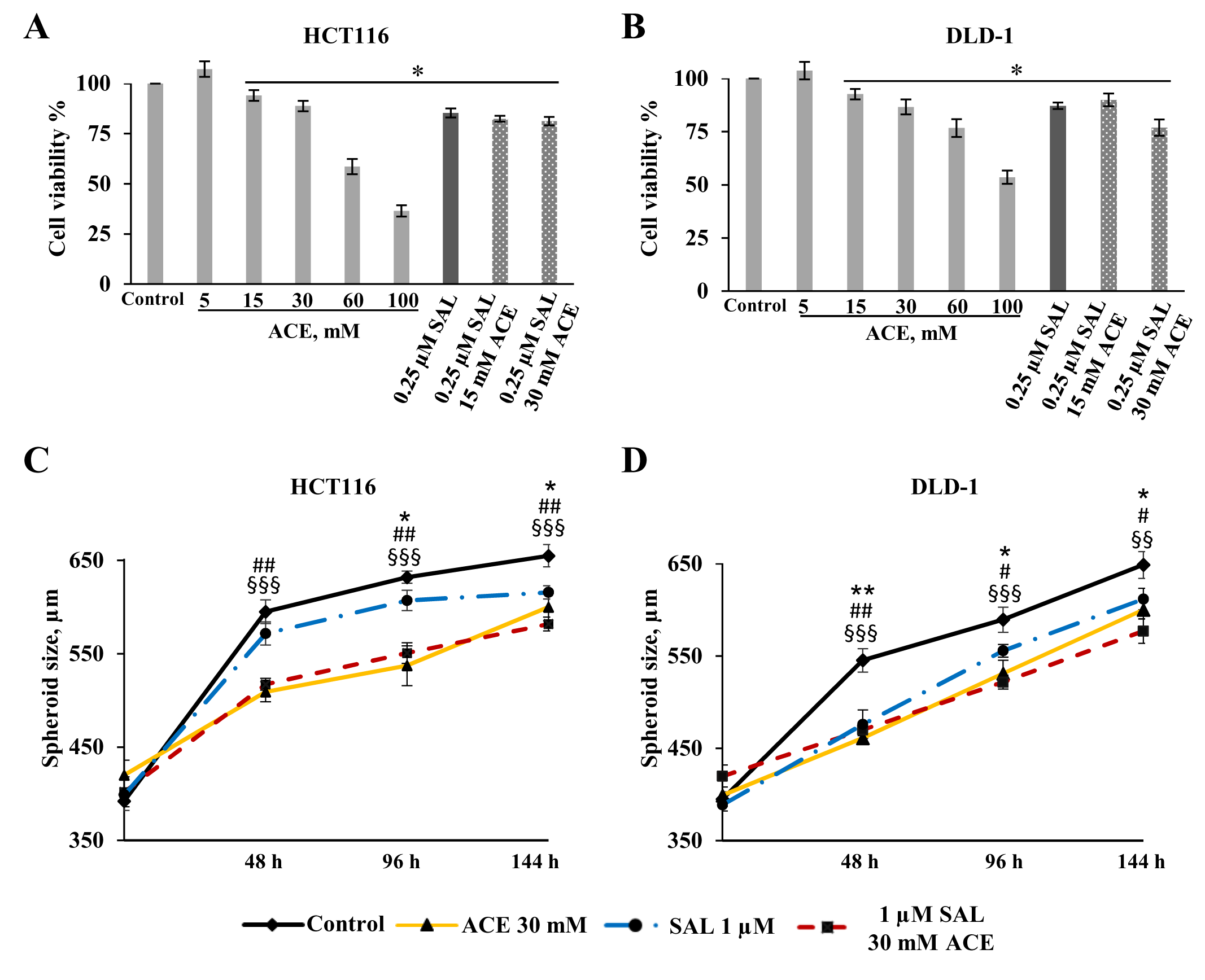
**

**Supplementary Figure 1. Cytotoxic effects of acetate (ACE) and salinomycin (SAL) in monotherapy and in combination on colorectal cancer cells in 2D and 3D cultures**.
**(A, B)** HTC116 and DLD-1 cell viability after 48-h treatment with ACE, SAL and their combination determined by the MTT assay. Chou-Talalay analysis of combinatory treatment with SAL and ACE did not show synergistic effect (determined CI values were > 1). **(C, D)** A time-response curve of cytotoxic effect of SAL, ACE and their combination on HCT116 and DLD-1 multicellular spheroids. Data are expressed as mean ± SEM calculated from 3 independent experiments (n = 3) measuring cell viability in 6 wells or 6 spheroid size, respectively, for each condition. * p < 0.05 (compared to control) for data in **A** and **B**. * p < 0.05, ** p < 0.01 (SAL compared to control); ^#^ p < 0.05, ^##^ p < 0.01, ^###^ p < 0.001 (ACE compared to control); ^§§^ p < 0.01, ^§§§^ p < 0.001 (SAL and ACE combination compared to control) for data in **C** and **D**.
